# Mulberry leaf reduces inflammation and insulin resistance in type 2 diabetic mice by TLRs and insulin Signalling pathway

**DOI:** 10.1186/s12906-019-2742-y

**Published:** 2019-11-21

**Authors:** Simin Tian, Min Wang, Chenyue Liu, Hongbin Zhao, Baosheng Zhao

**Affiliations:** 1 School of Chinese Materia Medica, Beijing University of Chinese Medicine, Beijing, 100029 China; 2 School of Chinese Medicine, Beijing University of Chinese Medicine, Beijing, 100029 China; 3 Beijing Research Institute of Chinese Medicine, Beijing University of Chinese Medicine, No.11 North 3rd Ring East Road, Chao-yang District, Beijing, 100029 China

**Keywords:** Diabetic mice, Mulberry leaf, Inflammation, Insulin resistance

## Abstract

**Background:**

It has been testified that Diabetes mellitus (DM) has a close association with chronic inflammation and Toll-like Receptors (TLRs), and DM could be prevented by mulberry leaf. Therefore, a hypothesis came into being that mulberry leaf could ameliorate proinflammation and insulin resistance (IR) through TLRs and insulin signalling pathways.

**Methods:**

Water extracts of mulberry leaf (WEM) was given to diabetic mice by gavage for 10 weeks, and the diabetic mice was injected with low-dose streptozocin, fed with high-fat and high-sugar diet. Oral glucose tolerance tests (OGTTs) were conducted. At the same time, homeostasis model assessment of insulin (HOMA-IR) and the level of the inflammatory factor, tumour necrosis factor-α (TNF-α) was measured. The expressions of critical nodes of TLRs and insulin signalling pathway were also examined.

**Results:**

WEM contributed to a significant decrease in fasting blood glucose, AUC from the investigation of OGTTs and HOMA-IR. The levels of the inflammatory factor, tumour necrosis factor-α (TNF-α) also declined. Moreover, WEM suppressed the expression of TLR2, myeloid differentiation primary-response protein 88 (MyD88), tumour-necrosis-factor receptor-associated factor 6 (TRAF6), nuclear factor kappa B (NF-κB) in the skeletal muscle. WEM could up-regulate the expression of insulin receptor (InsR) and insulin receptor substrate 1 (IRS1), and down-regulate the phosphorylation of IRS1 in adipose tissue.

**Conclusion:**

Through this study, a conclusion could be made that WEM mitigates hyperglycemia, IR, and inflammation through the interactions among TLR2 signalling pathway, insulin signalling pathway and TNF-α.

## Background

Mulberry leaf (*Morus alba* L.) has long been used as a traditional medicine or functional food to treat a variety of diseases since ancient China. In the light of food safety law in China, mulberry leaf has been listed in Edible Directory, which means that mulberry leaf could be used as daily food without any severe side effects reported, even in the long run. Mulberry leaf has been proved to inhibit oxidation, inflammation and diseases like atherosclerosis, Diabetes mellitus (DM), immunological disease and cancer could be preventable with mulberry leaf [1].

The incidence of DM, characterized by the metabolic syndromes and insulin resistance (IR), shows a booming growth in recent years [2]. The history of using mulberry tea to treat diabetes dates back to the sixteenth century, recorded in Compendium of Materia Medica (in Chinese “Ben CaoGang Mu”) by Li Shizhen. 1-dexoynojirimycin (DNJ), flavonoids and polysaccharides from mulberry leaf are validated to have hypoglycemic effect through inhibition of α-glucosidase [3]. Mulberry leaf extract could increase insulin sensitivity by activating phosphatidylinositol 3-kinase (PI3K)-protein kinase B (AKT) signalling pathway via the translocation of the glucose transporter 4 (GLUT4) to the plasma membrane and the increase in expressions of pancreatic duodenal homeobox-1 (PDX-1), insulin-1, and insulin-2 in the pancreas, and correct the imbalance of glucose metabolism by activating AMP-activated protein kinase (AMPK) signalling with the uptake of 3-O-methyl-D-glucose transport [4]. Mulberry leaf could attenuate IR by enhancing the expression of the peroxisome proliferator-activated receptor γ (PPARγ), and it also could reduce oxidative stress and suppress the apoptosis of β-cell and nuclear factor kappa B (NF-κB) signalling pathway to improve IR [4].

DM has been proved to have a close association with chronic inflammation and Toll-like Receptors (TLRs) [5]. TLRs display specific molecular patterns to mediate immune and nonimmune response, producing inflammatory cytokines including tumour necrosis factor-α (TNF-α) and interleukin-6 (IL-6) [6], which induces IR and suppresses insulin signal translocation [7]. Compared with normal controls, the disordered expressions of TLR2, TLR4, myeloid differentiation primary-response protein 88 (MyD88), tumour-necrosis-factor receptor-associated factor 6 (TRAF6), and NF-κB were observed in both DM patients and diabetic animals [8, 9], which suggests pro-inflammatory cytokines might play an important role in DM process.

Therefore, a speculation was formed that mulberry leaf could ameliorate inflammation and IR through TLRs and insulin signalling pathways; at the same time, there are few studies on the effect of mulberry on TLRs, so it might be a promising research to explore the new anti-diabetic mechanism and to develop a novel treatment with mulberry leaf.

Our previous researches have reported that mulberry leaf is able to down-regulate the expression of TLR2 and TLR4 in pancreas of KKAy [10], that the main components of water extracts of mulberry leaf (WEM) have been determined [11], and that mulberry leaf affect the TLRs mRNA in the liver of streptozocin (STZ)-induced diabetic mice [12]. Diabetic mice are used for the research, for they are induced by low dosage of STZ and high-fat and high-sugar diet, whose syndromes resemble the clinical disorders of DM. This study aims at evaluating how WEM affects TLRs and insulin signalling pathway in the skeletal muscle and adipose tissue where most changes in DM occur [13].

## Methods

### Preparation of WEM

Dried mulberry leaf was purchased from Tong Ren Tang Group Co., Ltd. in Beijing, China. The Chinese herb was identified as *Morus alba* L. by Professor Liu Chunsheng, Beijing University of Chinese Medicine. The mulberry leaf (300 g) were soaked in the deionized water (3.6 L) for 30 min, and extracted at 85–95 °C for 2.5 h. The concentrated extracts were placed into the vacuum oven and dried into powder. The powder was weighted, and the yield was calculated (yield: 15.95%) [11].

### Animals

Male ICR mice (18-20 g) were purchased from SPF (Beijing) Biology Co. Ltd. and were housed under laboratory animal barrier circumstance at Beijing University of Chinese Medicine. The mice were kept at 23 ± 2°C with a 60% relative humidity. They were offered 12 continuous hours of light each day. All experiments were performed in compliance with the relevant laws and institutional guidelines. The experimental procedures were approved by the Laboratory Animal Care Committee of Beijing University of Chinese Medicine (Approval Number: BUCM-4-2015072201-3002).

### Animal grouping, modeling and treatment

After 7 days of acclimatization, the mice were randomly allocated by weight at the ratio of 1:6 to a normal control group (NC, *n* = 12) offered with ordinary chow diet and a diabetic group (DG, *n* = 72) fed with high-fat and high-glucose diet. The high-fat and high-glucose diet was formulated by 20% sucrose, 2.5% cholesterol, 10% lard, 1% sodium cholate, and 66.5% chow diet. After a 10-week diet, all the mice were fasted overnight, and DG mice received intraperitoneal injection of STZ (Solarbio, China), freshly dissolved in cold buffer solution (0.1 M citric acid, pH 4.5) at 100 mg/kg for 3 days continuously, which were modified by the reference [14], while the NC mice were intraperitoneally injected with the buffer solution directly. After 5 days of injection, the DG mice, of which fasting glucose was > 12.0 mmol/L, were defined as diabetic mice for the following experiment.

According to the fasting glucose levels, the diabetic mice were randomly divided into 4 groups: (1) diabetic mice/not treated, (2) diabetic mice treated with 2 g/kg WEM, (3) diabetic mice treated with 4 g/kg WEM and (4) diabetic mice treated with 8 g/kg WEM. It is important to note that 2 g/kg, 4 g/kg and 8 g/kg stand for raw mulberry leaf, and that the dosages of the extracts are 0.319 g, 0.638 g and 1.276 g/kg respectively. The treated diabetic mice were given a certain amount of WEM by gavage once a day in the morning for 10 weeks. During the treatment, all the diabetic mice were kept with high-fat and high-sugar diet; the NC mice were fed with ordinary chow diet simultaneously.

The dose of extracts has been previously proved effective (data not shown) in lowering fasting glucose of diabetic mice. The fasting glucose and the fasting weight were measured after 12 h fasting once every 2 weeks. After 10 weeks of treatment and feed, all of the mice were anaesthetized by intraperitoneal injection with 50 mg/kg of 1% Pentobarbital Na (Sigma, USA) after fasting 12 h, and blood serums of the mice were collected from aortaventralis under anesthesia, which led to death due to hemorrhagic shock; pancreas, skeletal muscle and abdominal adipose tissue were quickly harvested, and then stored at − 80 °C for the subsequent experiment.

### Oral glucose tolerance tests (OGTTs)

In the 6th, 8th, and 10th weeks, OGTTs were conducted. After 12 h of fasting, the animals were administrated 1.5 g/kg glucose by gavage. Blood glucose was measured at 0, 15, 30, 60, 90, 120mins after glucose administration from the tail vein by glucometer [15].

### Hematoxylin and eosin (H&E) staining of pancreas

Pancreas from experimental mice was fixed in 10% neutral formalin for 72 h, then hydrated in decreasing concentrations of ethanol overnight, and embedded in paraffin. Sections were stained with H&E [16, 17].

### Measurement of plasma insulin, HOMA-IR, TNF-α, IL-6, and IL-1β

The plasma insulin (Alpco, USA), TNF-α (Life technologies, USA), IL-6 (Life technologies, USA) and IL-1β (Life technologies, USA) were measured by ELISA according to the instructions. Homeostasis model assessment of insulin resistance (HOMA-IR) was calculated as follows:

$$ \mathrm{HOMA}-\mathrm{IR}=\left(\mathrm{Fasting}\kern0.17em \mathrm{insulin}\times \mathrm{fasting}\kern0.17em \mathrm{glucose}\right)/22.5 $$

### Quantitative real-time reverse transcript-polymerase chain reaction (RT-qPCR) analysis

The specific sets of primers were designed by Primer express 3.0. Total RNA was isolated from skeletal muscle using TRIZOL (Invitrogen, USA). 2 μg total RNA was reverse-transcribed simultaneously into cDNA (Invitrogen, USA). The specific primers were listed in the following: TLR1, 5′-CCAACAGTCAGCCTCAAGCA-3′(forward),5′-CATATAGGCAGGGCATCAAAGG-3′(reverse);TLR2,5′-GAGGTGCGGACTGTTTCCTT-3′(forward);5′-GAGATTTGACGCTTTGTCTGAGG-3′(reverse);TLR3,5′-GGAGCCAGAACTGTGCCAAA-3′(forward),5′-GAGCTCATTATGTTGCAGGTTCA-3′(reverse);TLR4,5′-ACCTGGCTGGTTTACACGTC-3′(forward),5′-CTGCCAGAGACATTGCAGAA-3′(reverse);TLR5,5′-GCTGCAACTGGACCTTTCG-3′(forward),5′-CCCAAACAGTCGAGGATTCAA − 3′ (reverse); TLR6, 5′-GACCTCCACCAAGAACAAAAG-3′ (forward), 5′-GGCATCCGAAGCTCAGATATAGA-3′(reverse);TLR7,5′-TCTGGCCGTTGAGAGAGTTG − 3′ (forward), 5′-ATCAAGCCGGTTGTTGGAGA − 3′ (reverse); TLR8, 5′-TAGAAGTGCTGGACCTGAGC-3′(forward),5′-ATCCTAGACGGTGCGTTACC-3′(reverse);TLR9,5′-GCCTTCGTGGTGTTCGATAAG-3′(forward),5′-CACCCGCAGCTCGTTATACA-3′(reverse);β-actin,5′-GCAGGAGTACGATGAGTCCG-3′(forward),5′-CAGACTCAGTAACAGTCCGC-3′ (reverse). The expression of mRNA was measured using Power SYBR Master Mix (Applied Biosystems, USA) on CFX96 (Bio-Rad, CA, USA). The thermal cycling conditions started with Hold step at 95 °C for 10 min, followed by 40 cycles with denaturing at 95 °C for 15 s, and extended at 60 °C for 60 s.

Total RNA was extracted from adipose tissue, and cDNA was synthesized by 1 μg as described above. The cDNA was amplified utilizing TaqMan Gene expression Master Mix (Applied Biosystems, USA) on CFX96. The primers were directly purchased from Applied Biosystems: Insulin Receptor (InsR) (FAM, Mm01211875-ml), Insulin Receptor Substrate 1 (IRS1) (FAM, Mm01278327-ml), β-actin (VIC PL, Mm00607939-S1). The amplification conditions started with Hold step at 50 °C for 2 min and 95 °C for 10 min, followed by 40 cycles with denaturing at 95 °C for 15 s, and extended at 60 °C for 60 s.

Quantification of the mRNA was based on standard curves derived from cDNA standards for each primer pair. For normalization of differences in mRNA amounts, the housekeeping gene β-actin was co-amplified. The relative expression of target gene was calculated with Pfaffl.

### Western blot

The collected tissue was placed on the ice, then 100 μl of ice-cold lysate (PMSF: RIPA = 1:100) was added in the 10 mg of skeletal muscle, or 40 μl of ice-cold lysate (protein phosphatase inhibitor: PMSF: RIPA = 1:1:100) was added in the 10 mg of adipose tissue. The experimental objects were placed on the ice for 10 min, vortexed for 10 s, and stopped for 5 min for three times. The supernatant liquid was transferred, and stored at − 80 °C after the centrifugation at 4 °C, 12,000 g/min for 10 min. The protein concentration was carried out by BCA Protein Assay (Dingguo, Beijing, China). The protein lysate was mixed with 5 × SDS PAGE loading buffer (CWBIO, Beijing, China). The protein of adipose tissue was heated at 95 °C for 10mins, and protein of skeletal muscle was heated at 70 °C for 15mins. 20 μg total protein of adipose tissue and 40 μg total protein of skeletal muscle respectively were separated on 10% SDS-PAGE Gel and electrodotted to Polyvinylidene Fluoride (Millipore, USA) for 3 h at 200 V respectively. Membranes were blocked in Tris-buffered saline containing 0.1% Tween 20 (TBST) in 3% skim milk or 5% Bull Serum Albumin (BSA) for 1 h at room temperature, and then was incubated with the antibodies at 4°C overnight.

The following antibodies were for skeletal muscle: TLR1, 1:200 (Santa Cruz Biotechnology, CA), TLR2, 1:1000 (Abcam, Cambridge, UK); TRAF6, 1:2000 (Abcam, Cambridge, UK); MyD88, 1:500 (Abcam, Cambridge, UK); NF-κB p65, 1:1000 (Cell Signaling Technology, USA).

While for adipose tissue, these antibodies are needed: IRS1, 1:500 (Abcam, Cambridge, UK); InsR, 1:1000 (Abcam, Cambridge, UK); IRS1 (phospho), 1:1000(Abcam, Cambridge, UK).

Membranes were incubated with corresponding secondary antibodies at room temperature for 1 h after being washed with TBST. Proteins were imaged employing the efficient chemiluminescence kit (ECL) (GENVIEW, USA) on c600 Azure. All the membranes were re-probed with β-actin (1:5000, Proteintech, USA) for adipose tissue and α-tubulin (1:5000, Abcam, Cambridge, UK) for skeletal muscle respectively, used for the internal standard. ImageJ software (Java-based software program, National Institute of Health) was applied for the analyses.

### Statistical analysis

The area under curve (AUC) and total body clearance (CLTB) were calculated with DAS 3.2.0. All the results were expressed as mean ± SEM and examined with one-way analysis of variance (ANOVA) followed by Tukey’s comparisons. The significant differences were set at *P <* 0.05. SPSS 22.0 was utilized for calculation.

## Results

### The effect of WEM on body weight, blood glucose and insulin

Blood glucose at the beginning of treatment in the diabetic mice (23.13 ± 1.73 mmol/L) was much higher than that in normal mice (6.20 ± 0.31 mmol/L, *P <* 0.01). Fasting blood glucose of all the treatment groups with WEM reduced dramatically by the 6th week after the administration (*P <* 0.01), but the treatment with 8 g/kg WEM (5.28 ± 0.62 mmol/L) displayed the most significant reduction in fasting blood glucose compared with other diabetic mice (18.22 ± 2.37 mmol/L) (*P <* 0.05) till the end of the experiment (Table 1).

**Table 1:** Fasting blood glucose (mmol/L), and insulin (ng/ml) concentrations at the end of the trial

	Dosage	0wk	2wk	4wk	6wk	8wk	10wk	insulin
NC mice	–	6.20 ± 0.31	7.10 ± 0.55	6.62 ± 0.54	5.35 ± 0.58	3.90 ± 0.40	3.85 ± 0.40	0.52 ± 0.13
Diabetic mice	–	22.13 ± 1.73^**^	21.67 ± 7.41^*^	20.87 ± 3.80	16.60 ± 1.98^**^	16.03 ± 1.90^**^	18.22 ± 2.37^*^	0.17 ± 0.00
WEM	2 g/kg	22.95 ± 1.73	11.22 ± 2.89	9.57 ± 0.65	8.57 ± 1.25^##^	6.47 ± 0.89^#^	9.23 ± 1.29	0.25 ± 0.02^#^
	4 g/kg	21.95 ± 1.52	10.82 ± 3.25	11.70 ± 1.35	12.67 ± 1.88	6.87 ± 0.91^#^	9.43 ± 2.23	0.29 ± 0.03
	8 g/kg	22.12 ± 2.15	10.35 ± 4.52	9.85 ± 0.71	8.65 ± 1.59^##^	5.98 ± 0.79^#^	5.28 ± 0.62^#^	0.33 ± 0.04

Data are means ± SEM (*n* = 6).^*^P < 0.05, ^**^P < 0.01, NC mice vs. *Diabetic mice;* ^*#*^*P < 0.05,* ^*##*^*P < 0.01, WEM* vs. *Diabetic mice.* Fasting blood glucose (mmol/L) begins form 0wk from 10wk, and insulin (ng/ml) concentrations at the end of the trial is shown in the last column

**Fig. 1:**
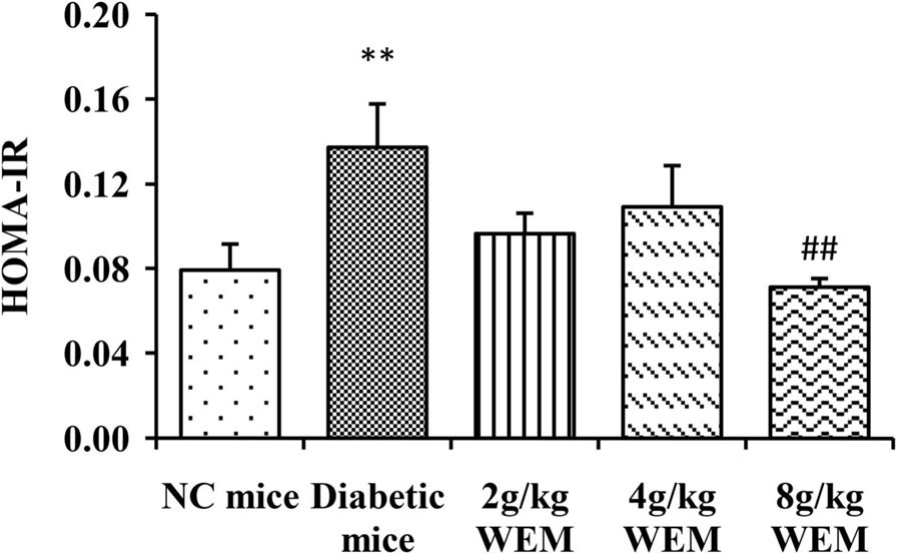
Homeostasis model assessment of insulin (HOMA-IR) at the end of the trial. Results are means ± SEM (*n* = 6). ^*^P < 0.05, ^**^P < 0.01, NC mice vs. *Diabetic mice;* ^*#*^*P < 0.05,* ^*##*^*P < 0.01, WEM* vs. *Diabetic mice*

Insulin level of the serum in 2 g/kg WEM mice (*P <* 0.05) significantly outnumbered that of the diabetic mice. It was concluded that the decrease in insulin of diabetic mice might be related with degree of islet cell deterioration, which influenced the secretion of insulin. When HOMA-IR was calculated (Fig. 1, Additional file 1: Table S1), there was a statistical decrease in the 8 g/kg WEM vs. diabetic mice (*P <* 0.01).

A tremendous decrease in body weight was detected in diabetic mice without any treatment, compared with NC mice in the 6th, 8th and 10th weeks (*P <* 0.01). WEM could elevate the body weights of the diabetic mice with WEM treatment, but for diabetic mice with no WEM treatment, there were no obvious changes in body weights (Table 2).

**Table 2:** Fasting Weight (g) Concentrations Following Treatment

	Dosage	0wk	2wk	4wk	6wk	8wk	10wk
NC mice	**–**	45.33 ± 2.60	46.17 ± 2.46	47.67 ± 0.49	48.17 ± 0.98	48.33 ± 0.71	48.50 ± 0.67
Diabetic Mice	**–**	45.50 ± 3.19	42.50 ± 1.82	43.67 ± 2.64	42.00 ± 0.63^**^	41.33 ± 1.43^**^	39.33 ± 1.52^**^
WEM	2 g/kg	46.50 ± 4.02	44.17 ± 3.94	43.83 ± 4.08	44.00 ± 3.75	46.50 ± 3.33	41.33 ± 2.79
	4 g/kg	45.00 ± 2.05	44.33 ± 1.96	44.50 ± 1.09	45.83 ± 1.35	43.83 ± 0.79	44.00 ± 1.86
	8 g/kg	45.83 ± 1.72	44.50 ± 1.95	43.33 ± 1.20	42.33 ± 1.28	43.33 ± 1.09	43.17 ± 0.79

Data are means ± SEM (*n* = 6).^*^P < 0.05, ^**^P < 0.01, NC mice vs. *Diabetic mice;* ^*#*^*P < 0.05,* ^*##*^*P < 0.01, WEM* vs. *Diabetic mice*

### The effect of WEM on OGTTs

**Fig. 2:**
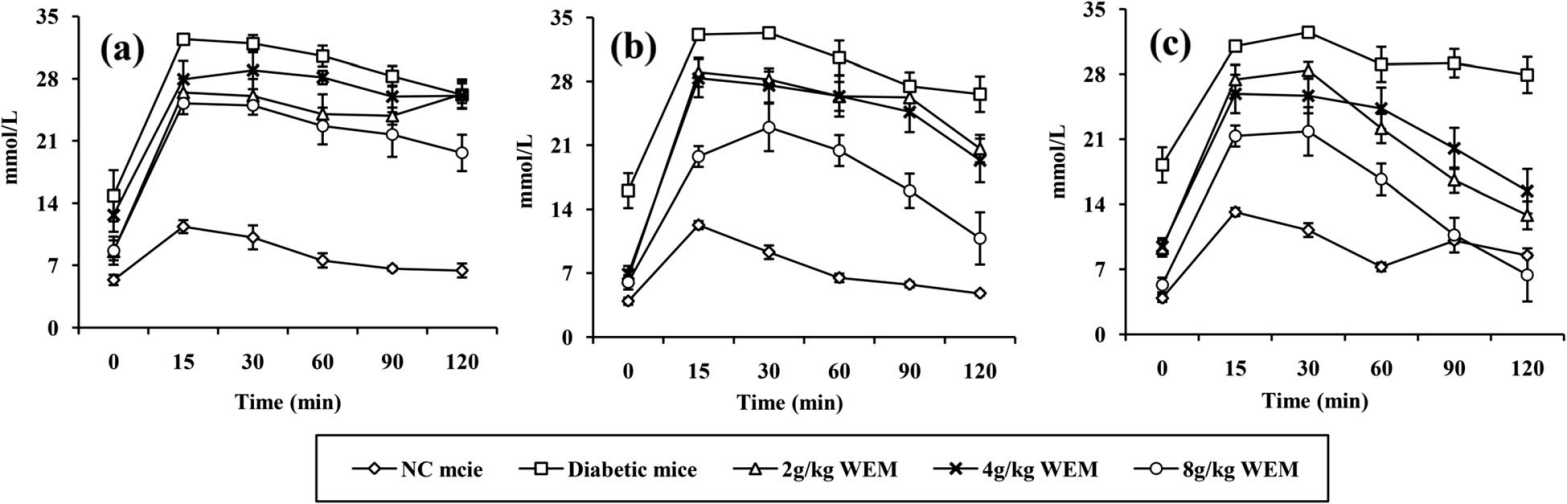
Blood glucose concentrations during the oral glucose tolerance tests (OGTTs) following the treatment for 6 (**a**), 8 (**b**) and 10 (**c**) weeks. Results are means ± SEM (*n* = 6)

To determine the effect of WEM on glucose tolerance, the OGTTs were performed in the 6th, 8th, and 10th week for comparison (Fig. 2, Additional file 1: Table S2-S4). During the whole experiment, three OGTTs were conducted in order to monitor when and how WEM played a role in fighting against diabetes. During OGTTs, an apparent difference was shown in blood glucose between the NC and diabetic mice at any time point (*P <* 0.05).

In the OGTTs (Fig. 2), the glucose level of NC mice rose rapidly, reaching to the peak at 15mins, whereas the blood glucose levels of diabetic mice increased sharply and remained stable between 15mins and 60mins. As the medication continued (Fig. 2c), a first-order kinetic of glucose elimination appeared for mice administered with 8 g/kg WEM.

**Fig. 3:**
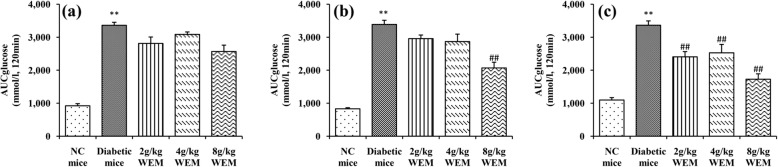
Area under the curve for glucose (AUC glucose) was calculated using DAS software in the 6th (**a**), 8th (**b**) and 10th (**c**) week starting from the treatment. Results are means ± SEM (*n* = 6). ^*^P < 0.05, ^**^P < 0.01, NC mice vs. *Diabetic mice;* ^*#*^*P < 0.05,* ^*##*^*P < 0.01, WEM* vs. *Diabetic mice*

In response to WEM, there was little difference in AUC glucose between diabetic mice and mice which received WEM (*P* > 0.05) after the 6-week treatment (Fig. 3a). However, in the following treatment, that is, in the 8th and 10th week, the 8 g/kg WEM AUC glucose was significantly lower than that of the diabetic AUC glucose (*P <* 0.01) till the end of the trial (Fig. 3b, c).

**Fig. 4:**
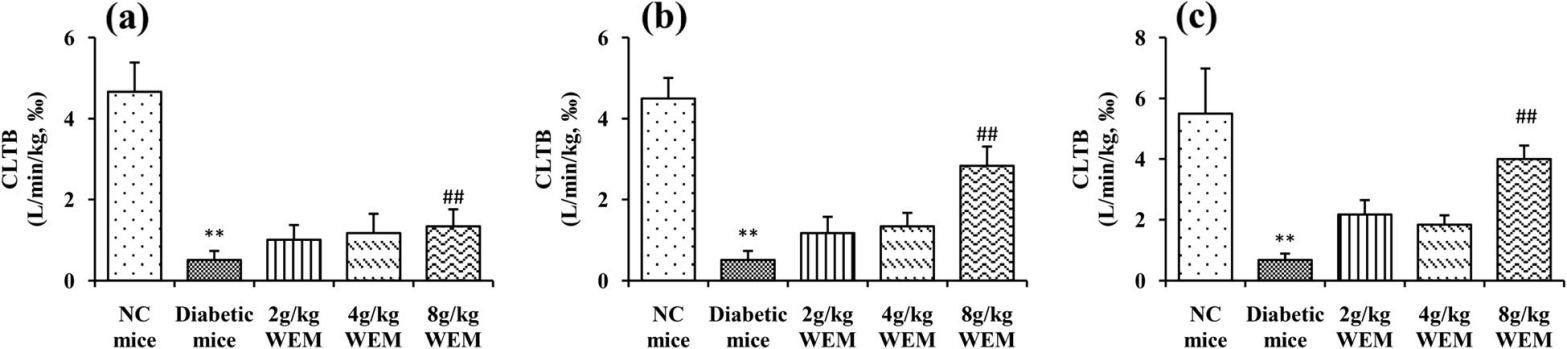
Total body clearance (CLTB) of glucose was calculated using DAS software in the 6th (**a**), 8th (**b**) and 10th (**c**) week starting from the treatment. Results are means ± SEM (*n* = 6). ^*^P < 0.05, ^**^P < 0.01, NC mice vs. *Diabetic mice;* ^*#*^*P < 0.05,* ^*##*^*P < 0.01, WEM* vs. *Diabetic mice*

In order to observe the clearance of glucose in the body, the index of CLTB was introduced, intending to find out whether there is a change in CLTB after the intervention of WEM. At the end of the trial, the diabetic CLTB (0.67 ± 0.21‰, *n* = 6) was notably lower than that of the NC mice (5.50 ± 1.48‰, *n* = 6). By contrast, 8 g/kg WEM could measurably increase the CLTB (4.00 ± 0.45‰, *n* = 6) compared with the diabetic mice (*P <* 0.01) (Fig. 4c).

### The effect of WEM on TNF-α, IL-1βand IL-6

**Fig. 5:**
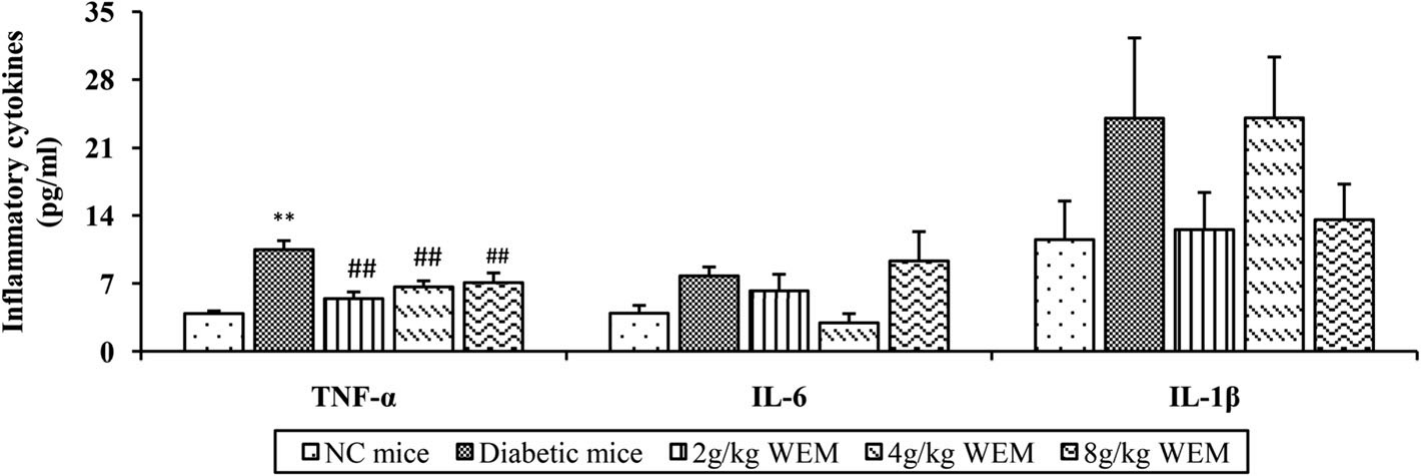
TNF-α, IL-1β and IL-6 were determined in the serum by ELISA. Bars represent means ± SEM (*n* = 5). *P < 0.05, **P < 0.01, NC mice vs. *Diabetic mice;* ^*#*^*P < 0.05,* ^*##*^*P < 0.01, WEM* vs. *Diabetic mice*

TNF-α, IL-1β and IL-6 are known to be up-regulated in DM, so those cytokines were assessed by ELISA. Although there were no statistical differences in IL-1β and IL-6 in the serum among all the experimental groups, it was observed that WEM had a little effect on lowering the concentrations of IL-1β and IL-6. TNF-α in diabetic mice (10.48 ± 0.98, *n* = 5) differed greatly from the NC mice (3.90 ± 0.27, *n* = 5) (*P <* 0.05), while WEM could dramatically reduce the concentration of TNF-α (*P <* 0.01) (Fig. 5, Additional file 1: Table S1).

### The effect of WEM on regulation of TLR mRNA expression and TLR protein in skeletal muscle

**Fig. 6:**
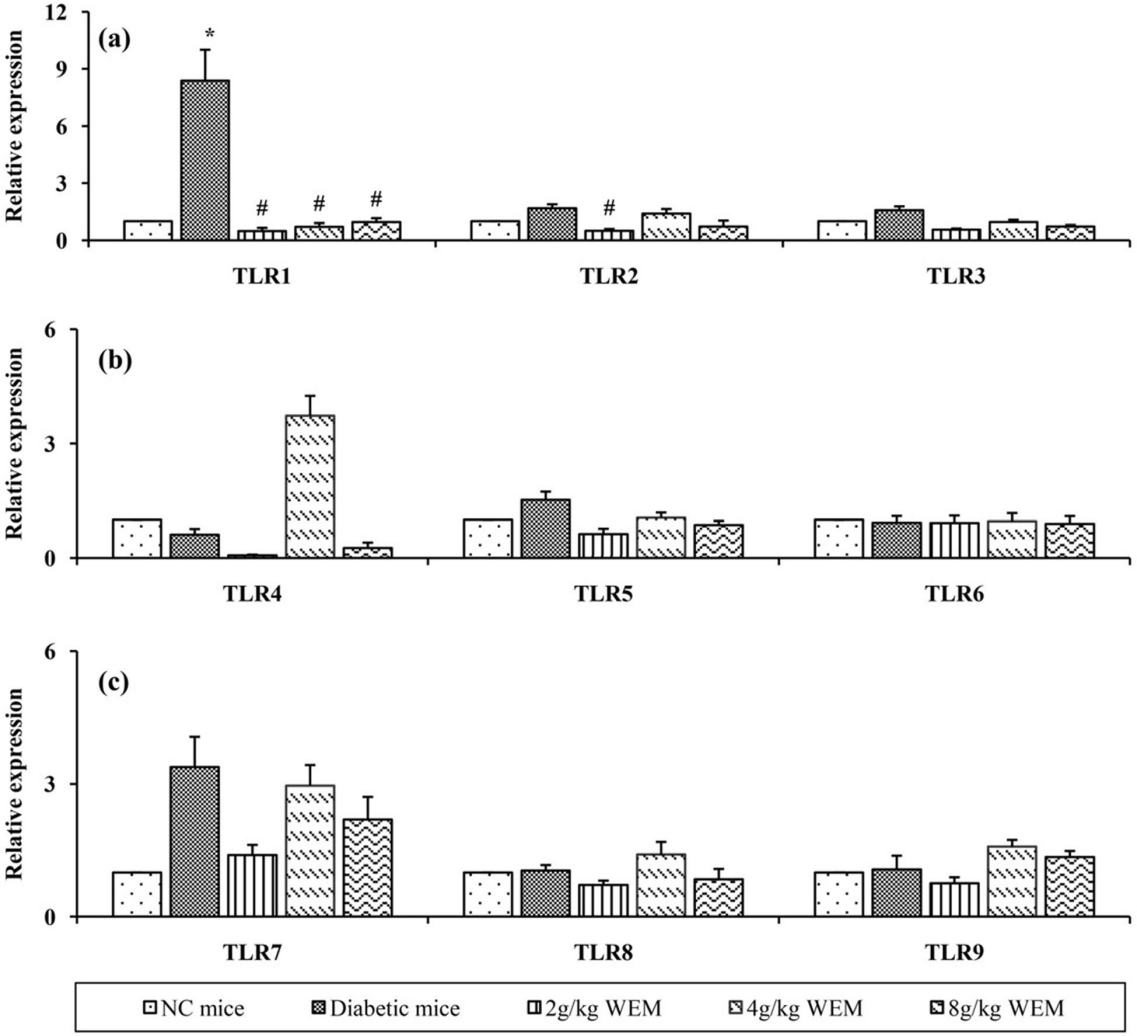
TLR mRNA relative expression pattern of skeletal muscle (**a**, **b** and **c**). RNA from skeletal muscle was isolated and quantitative real-time PCR (qPCR) was performed to assess the differences in TLR mRNA expression when the diabetic mice were administered with WEM. Data are shown as intensity ± SEM (*n* = 6to5). *P < 0.05, **P < 0.01, NC mice vs. *Diabetic mice;* ^*#*^*P < 0.05,* ^*##*^*P < 0.01, WEM* vs. *Diabetic mice*

To specify TLRs’ expressions in skeletal muscle after 10 weeks of treatment, mRNA was isolated from skeletal muscle, and quantitative Real-time PCR (qPCR) was performed for TLR1-TLR9. The expressions of TLR1, − 3 mRNA in diabetic mice were elevated with noticeable variation (*P <* 0.05) compared with the normal mice. Meanwhile, it was found that TLR1, − 2 mRNA after the treatment were statistically declined (*P <* 0.05) compared with the diabetic mice (Fig. 6, Additional file 1: Table S5 and S6), while WEM influenced the other TLR mRNA expressions marginally.

**Fig. 7:**
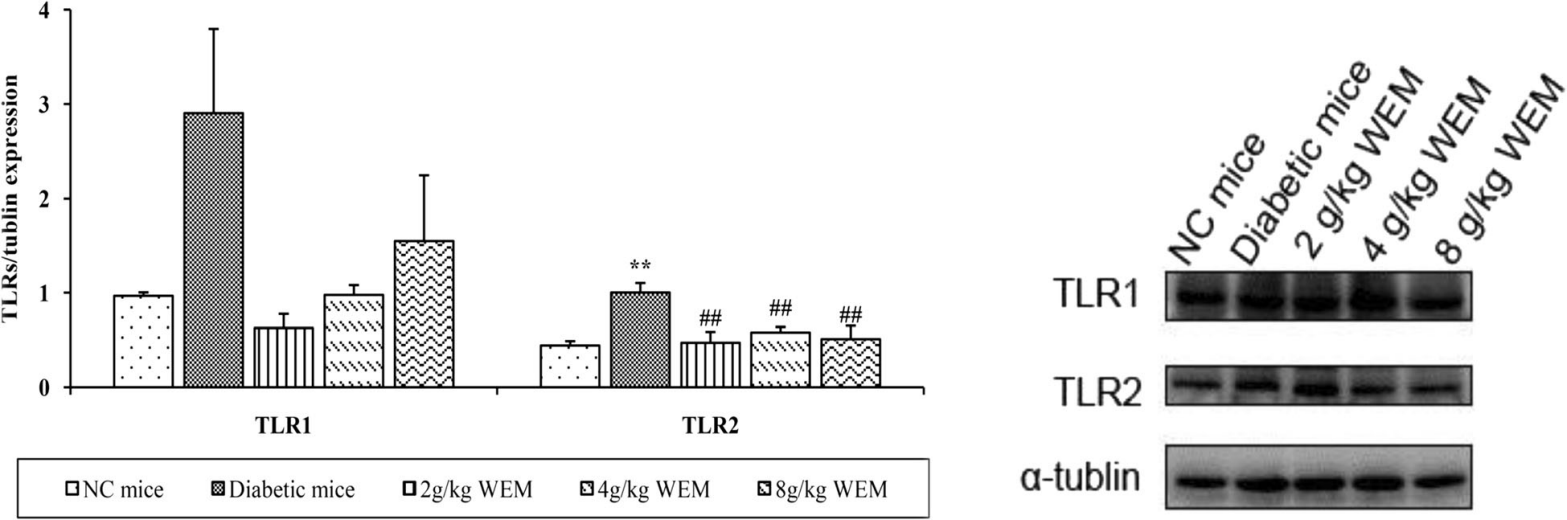
Effect of WEM treatment on protein expression TLR1 and TLR2. A significant decrease in TLR2 expression was observed with WEM. Normalized results are expressed as means ± SEM (*n* = 3). *P < 0.05, **P < 0.01, NC mice vs. *Diabetic mice;* ^*#*^*P < 0.05,* ^*##*^*P < 0.01, WEM* vs. *Diabetic mice*

To evaluate whether WEM affected the expression of TLRs on the protein level, western blot analysis was employed to further detect the presence of TLR1, − 2 protein. Western blot data revealed that TLR2 was elevated in the diabetic mice vs. in the NC mice (*P <* 0.01), and was dramatically reduced after the diabetic mice were administered with WEM (*P <* 0.01) (Fig. 7, Additional file: Table S7), but there was no evident difference in the protein expression of TLR1 between diabetic mice (not treated) and diabetic mice with WEM (*P* > 0.05) treatment (Fig. 7, Additional file: Table S7).

### The effect of WEM on TLR2 Signalling

**Fig. 8:**
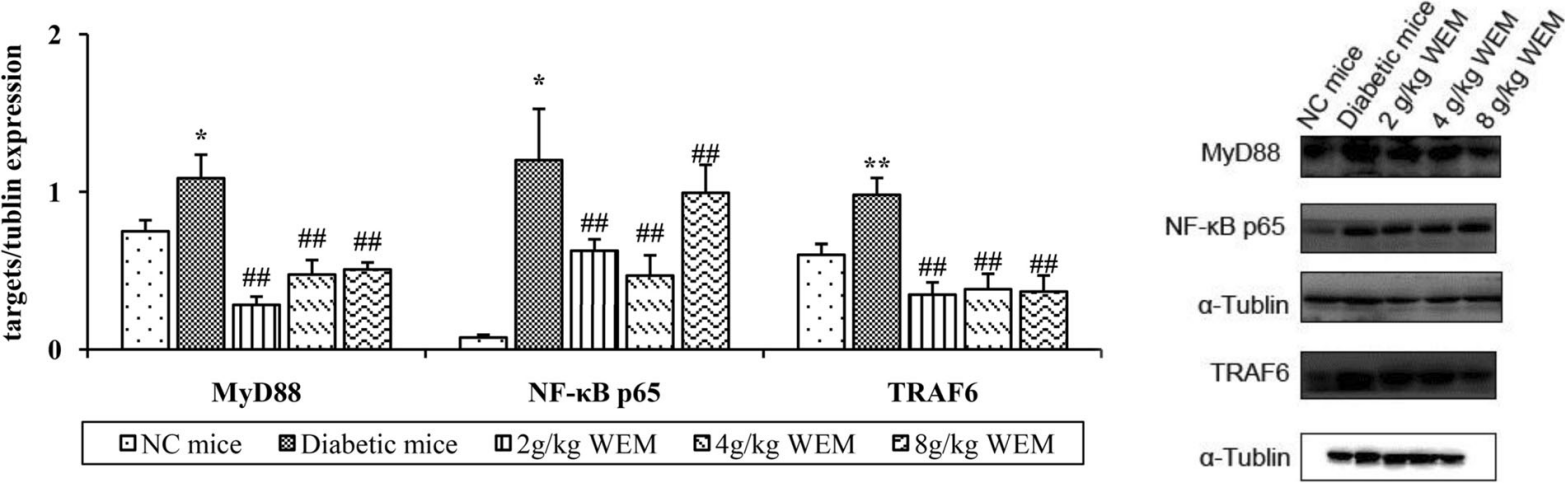
Downstream transcription factors’ expression interfered with WEM in diabetic mice. An evident decrease in MyD88 was detected after the treatment. According to the downstream transcription factor TRAF6 activation with WEM in diabetic mice, a profound decrease in TRAF6 was shown after the treatment. According to the downstream transcription factor NF-κB p65 activation with WEM in diabetic mice, NF-κB p65 expression showed a huge reduction after the treatment. Normalized results are expressed as means ± SEM (*n* = 3). *P < 0.05, **P < 0.01, NC mice vs. *Diabetic mice;* ^*#*^*P < 0.05,* ^*##*^*P < 0.01, WEM* vs. *Diabetic mice*

Apart from the role WEM played in down-regulating TLR2 expression, the critical messengers of TLR2 signalling were also monitored, such as MyD88, TRAF6 and NF-κB p65, to observe what impact they had on the signalling imposed by WEM. The expression of MyD88, TRAF6 and NF-κB p65 on the protein level were markedly higher in diabetic mice than in normal mice (Fig. 8, Additional file: Table S7). Furthermore, Western blot results demonstrated WEM could significantly inhibited the expressions of MyD88, TRAF6 and NF-κB p65 compared with the diabetic mice without treatment (*P <* 0.05) (Fig. 8, Additional file: Table S7).

### The effect of treatment on insulin Signalling pathway in adipose tissue

**Fig. 9:**
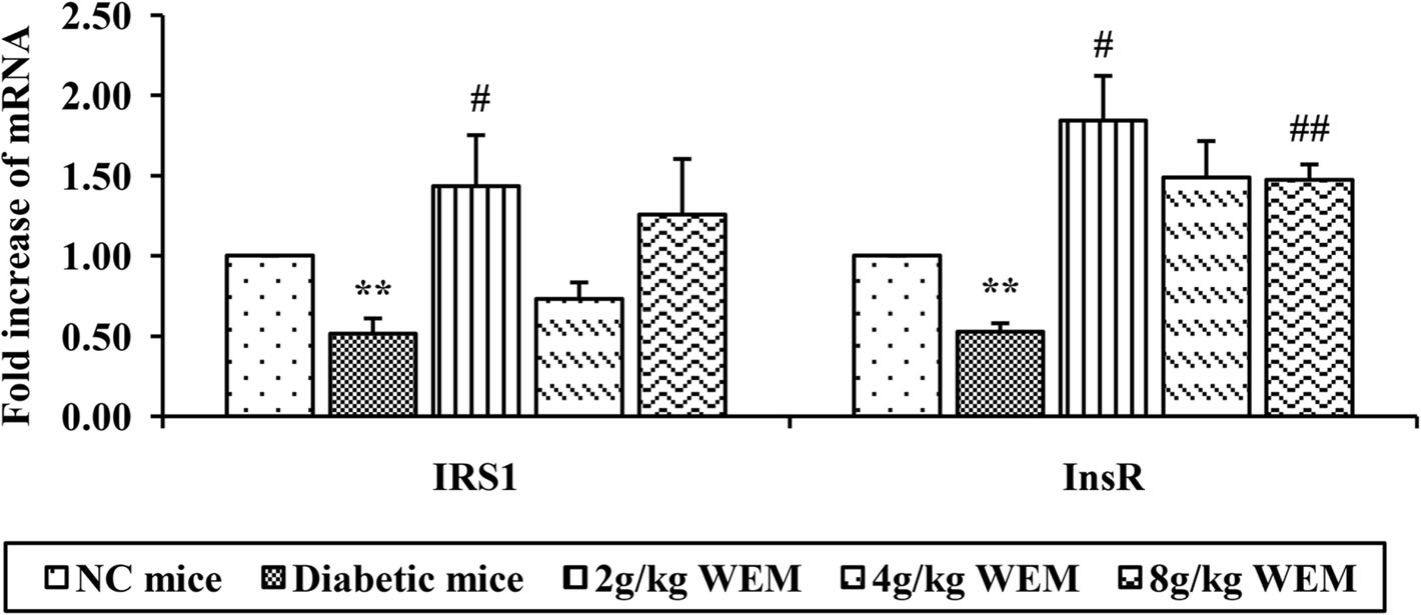
The gene expression of IRS1 and InsR affected by WEM in adipose tissue. WEM could increase the relative expression of IRS1 and InsR compared with diabetes. Data are shown as intensity ± SEM (*n* = 6to5). *P < 0.05, **P < 0.01, NC mice vs. *Diabetic mice;* ^*#*^*P < 0.05,* ^*##*^*P < 0.01, WEM* vs. *Diabetic mice*

To further test the ability of WEM in modulating insulin signalling pathway, IRS1, InsR, and phosphorylated IRS1 were utilized as the critical messengers. Initially, qPCR was performed to detect the gene expression of IRS1 and InsR. It was demonstrated that, for diabetic mice, the expression of the two genes were dramatically inhibited, while for mice with WEM treatment (*P <* 0.01), their gene expressions were greatly elevated (*P <* 0.05) (Fig. 9, Additional file: Table S8).

**Fig. 10:**
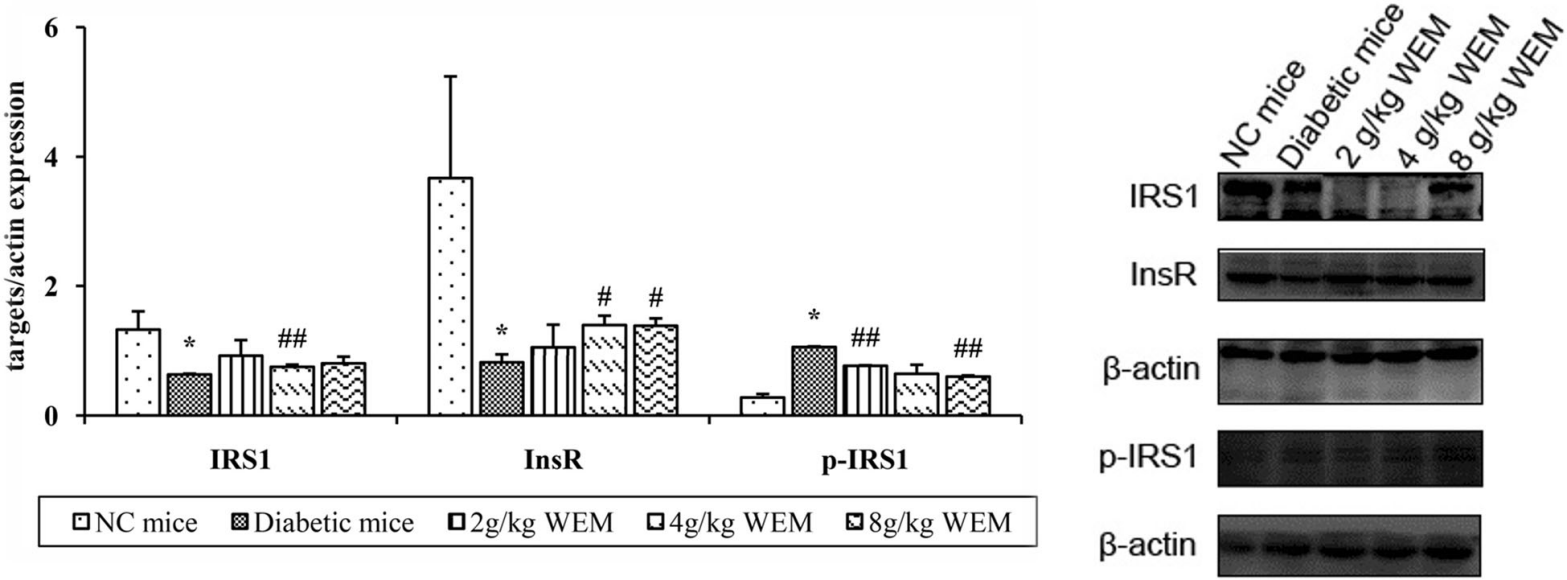
Protein expression of IRS1 and InsR influenced by WEM in adipose tissue of diabetic mice. A significant elevation of IRS1 and InsR was observed. The effect of WEM on phosphorylation of IRS1 (p-IRS1). An obvious reduction in p-IRS1 was observed among the treatment groups. Data are shown as means ± SEM (*n* = 3). *P < 0.05, **P < 0.01, NC mice vs. *Diabetic mice;* ^*#*^*P < 0.05,* ^*##*^*P < 0.01, WEM* vs. *Diabetic mice*

To investigate the expressions of IRS1, InsR and phosphorylated IRS1 on the protein level and confirm the results from above experiments, western blot analysis was introduced. It is shown that the protein expressions of IRS1, InsR were up-regulated in the following treatment with WEM (*P <* 0.05) (Fig. 10, Additional file: Table S9), suggesting the results were in accordance with the qPCR data. More importantly, it was found that WEM could greatly down-regulate IRS1 phosphorylation of the diabetic mice, compared with those without WEM treatment (*P <* 0.01) (Fig. 10, Additional file: Table S9).

### The effect of WEM on pancreas

**Fig. 11:**
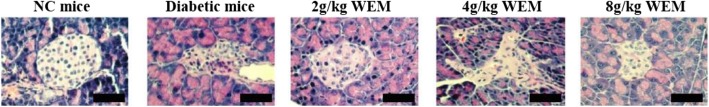
H&E staining of pancreas in experimental mice at the 10-week administration. Scale bars, 200 μm

To probe into the effect of WEM on pancreas, the inflammatory cells abounded in the pancreas of the diabetic mice were also observed at the end of the experiment, in comparison with the NC mice. After 10 weeks of treatment using WEM, morphological defects in the islet of the mice which received 8 g/kg WEM were alleviated, in terms of the number of inflammatory cells (Fig. 11). The change in the morphology of pancreases happened to diabetic mice with WEM treatment displayed that WEM could reduce the inflammatory status to some degree.

## Discussion

Mulberry leaf, as a kind of medicinal and edible material, has various supplementary functions in China. Experiments by Andallu [18], and Nalk [19] also proved that mulberry leaf could improve the diabetic symptoms of excessive food consumption, body weight, fasting glucose, urine sugar, glycosylated hemoglobin, triglyceride, cholesterol, and the few of β cells, etc., in the meantime, there was no side effect observed in the normal rats fed with mulberry leaf. Up to now, mulberry leaf could possibly make the gastrointestine malfunction in clinical experiments, due to the alpha glucosidase inhibition, a mechanism of lowering blood glucose with mulberry leaf [20]. WEM was proved to be hypoglycemic in line with other similar studies.

*Morus alba* L. is a kind of plant rich in phytochemicals, whose extracts could be used to treat various maladies [21]. Currently, some of the chemicals in the extracts of *Morus alba* L. have been isolated, purified and proved bioactive in diabetes, and they are alkaloids, such as 1-DNJ; flavonoids, such as rutin, quercetin, isoquercetin, and dihydronaphthyl; and polysaccharides mainly composed of mannose, rhamnose, glucose, xylose and arabinose. WEM was chosen as the agent to explore the synergic mechanism of anti-diabetes in STZ-induced mice with high-fat and high-sugar diet, because it suits the popular usage habit. In the previous studies, the main compounds in WEM have been identified, as well as the main metabolites in the serum of normal rats administered with WEM [11], aiming to provide bioactive substances for the subsequent research. WEM mainly consists of 11 chemicals. Some of them are glycoside compounds and others flavonoids, most of which have been proved to play pivotal roles in lowering the blood glucose [11].

The study was focused on high-fat and high-sugar diet-fed mice with intraperitoneal injection of STZ, which resulted in hyperglycemia, hypoinsulinaemia and noticeable deterioration of glucose elimination. The described syndromes indicated IR and islet β-cell dysfunction, suitable for the study on DM [22].

Controlling glycemic levels to the normal range is a top priority for the treatment of DM, because it could ameliorate metabolic abnormality and prevent the body from diabetic complications [23]. The glucose levels decreased after 6 weeks of treatment with WEM. Diabetic mice with 8 g/kg WEM (71%) treatment displayed a faster decline of glucose than those with 2 g/kg WEM (49%) and 4 g/kg (48%) treatment in terms of time and dose. The results were in accordance with other similar studies involving diabetic animal models treated with mulberry leaf [24].

Glucose intolerance is a typical feature of DM, attributed to IR and islet β-cell dysfunction. The glucose test is conducted to figure out if a mouse is diabetic or glucose intolerance [25]. During the whole experiment, three OGTTs were conducted in order to find out when and how WEM played a role in fighting against diabetes. The data showed that the blood glucose levels of mice with 8 g/kg WEM treatment decreased at 60mins, whereas the glycemic levels in diabetic mice fell at a slower rate when the blood glucose levels both sharply climbed to the peak level at 15mins. Therefore, visible differences were detected in the AUC glucose curve between 8 g/kg WEM and diabetic mice. Furthermore, 8 g/kg WEM could reduce HOMA-IR dramatically compared with the diabetic mice. These results highlighted that WEM could enhance insulin sensitivity. It was observed that the glucose clearance rate in mice treated with WEM was significantly higher than that in diabetic mice, indicating that WEM could improve the glycometabolism.

An abundance of evidence has reported that the pathophysiological process of diabetes has a close link among metabolism, systemic inflammation and innate immune response based upon large amounts of experimental animal models and clinical practices [7, 26]. TLRs play detrimental roles in activating inflammatory response, initiating adaptive immune response and stimulating the subsequent inflammatory reactions [27].

At least 13 TLRs have been identified in humans and mice, except for TLR 10, − 12 and − 13, and the other TLRs have their own ligands and unique biological roles [28], so the gene expression of TLR1-TLR9 was analyzed to find out which of TLRs could be regulated by WEM. The results showed that both gene and protein expression of TLR2 increased in the diabetic mice, which was similar to the relative results [29].

TLR2 is critical in the pathogenesis of IR, inflammation and diabetes in both clinics and experiments [30]. TLR2 expression arises in the Type 2 diabetes patients [6], and the TLR2 −/− knockout model. It is shown that TLR2 senses β-cell death and contributes to diabetes [31], and concluded that the deficiency of TLR2 decrease the diabetes-induced inflammation. The hyperglycemia could elevate the expression of TLR2 mRNA in a time-dependent manner [32]. In the experiment, the expression of TLR2 in diabetic mice was evidently higher than that in the NC mice, after the treatment, it was found that WEM could markedly inhibit the expression of TLR2; therefore, a conclusion was drawn that WEM fought against inflammation and lowered glucose through inhibition of TLR2 signalling pathway.

TLR2 signals via MyD88, leading to the activation of transforming growth factor-β-activated kinase 1, which via TRAF6, subsequently induces NF-κB [33], resulting in inflammatory expression. When TLR2 was knockdown under diabetic conditions, there was a sharp reduction in NF-κB activity and MyD88 expression [32]. The expressions of those described transcription factors increased in the diabetic, suggesting that the diabetic mice were in the inflammatory condition and suitable for the subsequent pharmacological research. As expected, the findings proved that WEM could inhibit the activation of MyD88, interfere with the expression of TRAF6, and decrease the transactivation of NF-κB. Therefore, WEM neutralized the inflammation and high glucose possibly through TLR2 signalling.

IR is a common pathological state of DM [34], in which normal concentration of insulin represents the abnormal response. This pathophysiology involves insulin-signalling network [35]. InsR and the insulin receptor substrate (IRS) are the critical nodes for the insulin action in the signalling [35]. The down-regulated InsR at the protein level is linked to the IR state in regard to DM [36]. IRS proteins have six isoforms in the family, with its emphasis on IRS1, because IRS1 has wide distributions and its deficiency leads to IR and glucose intolerance [37]. IRS1 phosphorylation responses to insulin and cytokines, like TNF-α and IL-6, and negatively regulates the insulin signalling pathway, resulting in IR [38]. All in all, the decrease in IRS1 and InsR cause IR in diabetic human and diabetic rodent animals [39]. The expressions of IRS1, InsR, and the phosphorylation of IRS in diabetic mice of the experiment conformed to the aforementioned description. Fortunately, after 10 weeks of treatment, WEM could measurably elevate the expression of IRS1 and InsR, and reduce the IRS phosphorylation, illustrating that WEM alleviates IR and diabetic status through insulin signalling pathway.

TNF-α is the first inflammatory cytokines discovered, which shows a close association with IR [40]. TNF-α-activating signalling pathway could interfere with insulin signalling pathway through interactions [35]. Also, TLRs are potentially targeted at mediating the cytokines' response [41, 42], and Stamm [43] reported that TLR2 and TLR4 regulate the TNF-α response. The results indicated that WEM profoundly inhibited the production of TNF-α in the serum in comparison with diabetic mice and could abate the inflammatory state and IR in the diabetic mice. It is found that mulberry leaf could be developed as an anti-diabetic supplementary by suppressing inflammation and IR.

Apart from the achievements above, the research has some limitations. Firstly, the expressions of TNF-α in the muscle and adipose tissue were not testified, though its concentration in the serum were tested, which could represent that the whole body was in the status of inflammatory condition. Then, a quantitative analysis might be needed for the determined components of WEM for the subsequent exposure experiment. Future studies may get the focus on the connections among TNF-α, TLR2 signalling and insulin pathways imposed upon WEM under the diabetic condition. Thirdly, fasting blood glucose was witnessed to fluctuate between the 8th and the 10th week in the treatment, which may be affected by the internal environment of blood glucose, the length of fasting time and so on. The subsequent experiments should minimize the error by strictly controlling the procedure. Next, the side-effects of mulberry leaf on NC mice were not monitored, because it was reported that mulberry leaf has little effects on normal rats. The side effects of WEM on the NC mice might be investigated in the future experiment for the comprehensive and credible results. Lastly, some results appeared no dose-dependent manner, which could result from the complex ingredients of WEM, a problem that always plagues researches of Chinese Medicine, so in the subsequent future, we will try to solve the problem through isolating the ingredients further.

## Conclusion

In conclusion, the study demonstrates that WEM have profoundly improved the damaged glucose tolerance, alleviate inflammation and mend IR. It is most likely that the main mechanisms of anti-diabetes involve suppressing TLR2 signalling pathway, stimulating the insulin signalling pathway and the interactions of the two through TNF-α based upon the bioactive compounds identified from WEM. Thus, WEM shows the potentiality of supplementary therapeutic strategy, aiming at decreasing TLRs to abrogate inflammation and lowering the risk of diabetes and complications.

## Additional file

**Additional file 1.** **Table S1.** Homeostasis model assessment of insulin (HOMA-IR),TNF-α, IL-1β and IL-6 at the end of the trial. **Table S2.** Blood glucose concentrations during the oral glucose tolerance tests (OGTTs) following the treatment for 6 weeks. **Table S3.** Blood glucose concentrations during the oral glucose tolerance tests (OGTTs) following the treatment for 8 weeks. **Table S4.** Blood glucose concentrations during the oral glucose tolerance tests (OGTTs) following the treatment for 10 weeks. **Tables S5** and **S6** TLR mRNA relative expression pattern of skeletal muscle. **Table S7** and the original western blots Effect of WEM treatment on protein expression TLR1, TLR2, and the downstream transcription factors' expression. **Table S8.** The gene expression of IRS1 and InsR affected by WEM in adipose tissue. **Table S9** and the original western blots Protein expression of IRS1 and InsR influenced by WEM in adipose tissue of diabetic mice.

## Data Availability

The datasets generated during and/or analysed during the current study are available from the corresponding author on reasonable request.

## Abbreviations

AMPK: AMP-activated protein kinase
DM: Diabetes mellitus
DNJ: 1-dexoynojirimycin
HOMA-IR: Homeostasis model assessment of insulin resistance
IL-6: Interleukin-6
InsR: Insulin receptor
IR: Insulin Resistance
IRS1: Insulin receptor substrate 1
MyD88: Myeloid differentiation primary-response protein 88
NF-κB: Nuclear factor kappa B
OGTTs: Oral glucose tolerance tests
PI3K: Phosphatidylinositol 3-kinase
PMSF: Phenylmethanesulfonyl fluoride
PPARs: Peroxisome proliferator-activated receptors
RT-q*PCR*: Quantitative real-time reverse transcript-polymerase chain reaction
STZ: Streptozocin
TLRs: Toll-like Receptors
TNF-α: Tumour necrosis factor-α
TRAF6: Tumour-necrosis-factor receptor-associated factor 6
WEM: Water extracts of mulberry leaf
