# Supplementary material for: Mulberry leaf reduces inflammation and insulin resistance in type 2 diabetic mice by TLRs and insulin Signalling pathway
Source: BMC Complement Altern Med. 2019 Nov 21;19:326. doi: 10.1186/s12906-019-2742-y (PMC6873489; doi:10.1186/s12906-019-2742-y)

Table S1 Raw data of HOMA-IR, TNF-α, IL -6, IL-1β

	Dosage	HOMA-IR	TNF-α(ng/ml)	IL-6(ng/ml)	IL-1β(ng/ml)
NC mice	-	0.08±0.01	3.90±0.27	3.93±0.82	11.53±3.97
Diabetic Mice	-	0.14±0.02	10.48±0.94	7.77±0.94	24.00±8.28
WEM	2g/kg	0.10±0.02	5.45±0.68	6.26±1.70	12.54±3.84
	4g/kg	0.11±0.02	6.65±0.63	2.93±0.96	24.03±6.29
	8g/kg	0.07±0.00	7.07±1.01	9.32±3.03	13.56±3.69

Data are means ± SEM (n=5).

Table S2 Raw data of OGTT in the 6th week (mmol/L)

	Dosage	0min	15min	30min	60min	90min	120min
NC mice	-	5.35±0.58	3.90±0.27	3.93±0.82	7.55±0.80	6.63±0.27	6.42±0.79
Diabetic Mice	-	14.82±2.87	32.42±0.40	31.92±0.97	30.53±1.16	26.18±1.57	22.97±2.04
WEM	2g/kg	8.57±1.25	26.43±1.56	26.05±1.91	24.02±2.22	23.82±3.38	26.25±1.67
	4g/kg	12.67±1.88	27.93±2.04	28.93±2.11	28.13±0.75	25.98±1.24	26.08±1.39
	8g/kg	8.65±1.59	25.22±1.22	24.98±1.04	22.67±2.07	21.70±2.52	19.63±2.05

Data are means ± SEM (n=5).

Table S3 Raw data of OGTT in the 8th week (mmol/L)

	Dosage	0min	15min	30min	60min	90min	120min
NC mice	-	3.90±0.40	12.27±0.45	9.28±0.74	6.47±0.47	5.73±0.31	4.77±0.24
Diabetic Mice	-	16.03±1.90	33.12±0.18	33.30±0.00	30.58±1.89	27.47±1.53	26.60±1.94
WEM	2g/kg	6.47±0.89	29.00±1.59	28.20±0.90	26.37±1.59	26.23±1.35	20.63±1.51
	4g/kg	6.87±0.91	28.35±2.08	27.57±1.87	26.40±2.25	24.67±2.22	19.33±2.39
	8g/kg	5.98±0.79	19.77±1.14	22.97±2.61	20.42±1.70	16.02±1.87	10.80±2.87

Data are means ± SEM (n=5).

Table S4 Raw data of OGTT in the 10th week (mmol/L)

	Dosage	0min	15min	30min	60min	90min	120min
**NC mice**	**-**	3.85±0.40	13.17±1.86	11.22±1.27	7.23±0.37	10.08±0.70	8.47±0.54
**Diabetic Mice**	**-**	18.22±2.37	31.00±1.30	32.50±0.53	29.05±1.90	29.18±1.53	27.92±1.88
**WEM**	**2g/kg**	9.23±1.29	27.42±1.81	28.42±0.79	22.15±2.03	16.58±1.87	12.80±2.50
	**4g/kg**	9.43±2.23	25.88±2.29	25.67±2.94	24.32±2.67	20.00±2.08	15.40±1.66
	**8g/kg**	5.28±0.62	21.33±2.15	21.83±1.65	16.67±1.65	10.67±1.80	6.37±1.16

Data are means ± SEM (n=5).

Table S5 Raw data of gene expression of muscle (1)

	Dosage	TLR1	TLR2	TLR3	TLR4	TLR5
**NC mice**	**-**	1.00±0.00	1.00±0.00	1.00±0.00	1.00±0.00	10.08±0.00
**Diabetic Mice**	**-**	8.38±1.61	1.69±0.20	1.57±0.21	0.61±0.15	1.52±0.22
**WEM**	**2g/kg**	0.49±0.18	0.50±0.10	0.56±0.07	0.07±0.02	0.63.±0.14
	**4g/kg**	0.72±0.18	1.40±0.24	0.95±0.13	3.73±0.52	1.06±0.13
	**8g/kg**	0.95±0.21	0.74±0.29	0.74±0.06	0.27±0.14	0.86±0.11

Data are means ± SEM (n=5-6).

Table S6 Raw data of gene expression of muscle (2)

	Dosage	TLR6	TLR7	TLR8	TLR9
**NC mice**	**-**	1.00±0.00	1.00±0.00	1.00±0.00	1.00±0.00
**Diabetic Mice**	**-**	0.92±1.61	3.38±0.69	1.04±0.13	1.07±0.31
**WEM**	**2g/kg**	0.91±0.18	1.40±0.23	0.72±0.10	0.76±0.14
	**4g/kg**	0.96±0.18	2.96±0.47	1.40±0.29	1.58±0.15
	**8g/kg**	0.89±0.21	2.20±0.51	0.85±0.23	1.35±0.14

Data are means ± SEM (n=5-6).

Table S7 Raw data of protein expression of muscle

	Dosage	TLR1	TLR2	MyD88	NF-κB p65	TRAF 6
**NC mice**	**-**	0.97±0.04	0.44±0.05	0.75±0.07	0.08±0.02	0.60±0.07
**Diabetic Mice**	**-**	2.91±0.89	1.01±0.10	1.09±0.15	1.20±0.33	0.98±0.11
**WEM**	**2g/kg**	0.63±0.15	0.47±0.11	0.28±0.05	0.63±0.07	0.35±0.08
	**4g/kg**	0.98±0.11	0.58±0.06	0.48±0.09	0.47±0.13	0.38±0.10
	**8g/kg**	1.55±0.69	0.51±0.14	0.51±0.04	0.99±0.18	0.37±0.10

Data are means ± SEM (n=3).

The original western blots are listed below.

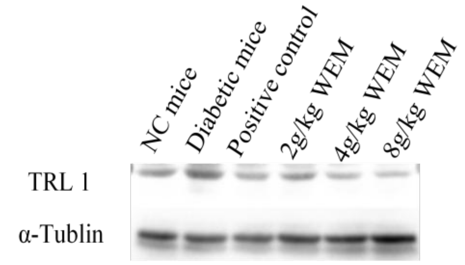

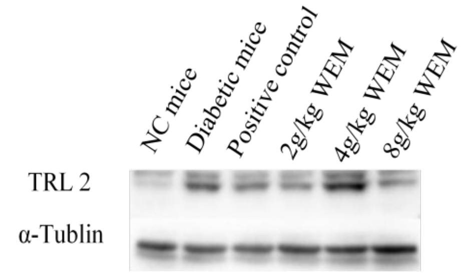

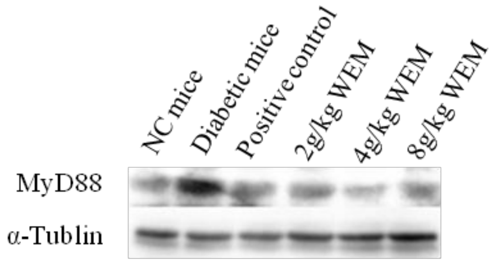

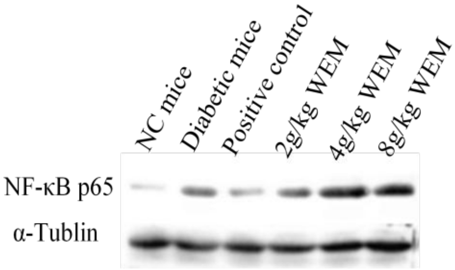

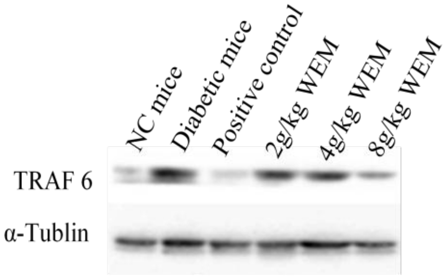

Table S8 Raw data of gene expression of fat

	Dosage	IRS1	InsR
**NC mice**	**-**	1.00±0.00	1.00±0.00
**Diabetic Mice**	**-**	0.52±0.10	0.53±0.05
**WEM**	**2g/kg**	1.44±0.32	1.84±0.28
	**4g/kg**	0.73±0.10	1.49±0.23
	**8g/kg**	1.26±0.35	1.47±0.10

Data are means ± SEM (n=3).

Table S9 Raw data of protein expression of fat

	Dosage	IRS1	InsR	p-IRS1
**NC mice**	**-**	1.33±0.28	3.67±1.57	0.28±0.06
**Diabetic Mice**	**-**	0.63±0.01	0.82±0.12	1.06±0.01
**WEM**	**2g/kg**	0.92±0.24	1.06±0.35	0.77±0.01
	**4g/kg**	0.75±0.03	1.40±0.14	0.65±0.14
	**8g/kg**	0.80±0.10	1.39±0.11	0.60±0.02

Data are means ± SEM (n=3).

The original western blots are listed below.

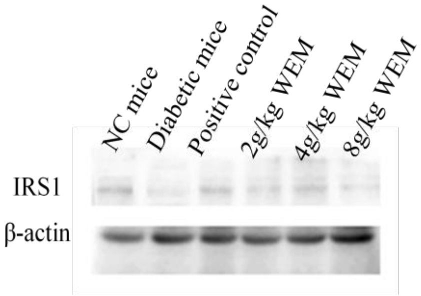

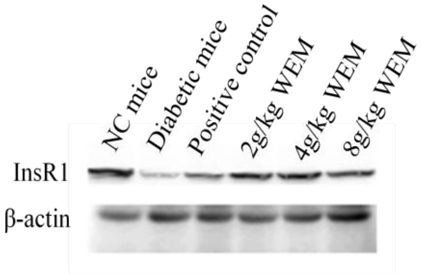

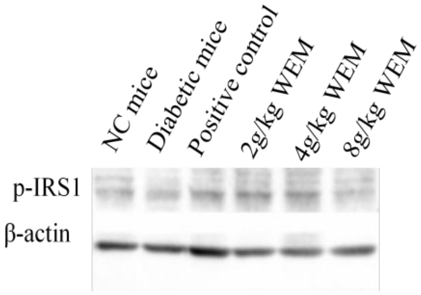

Supplement: Supplementary file 1 — Additional file 1. Table S1. Homeostasis model assessment of insulin (HOMA-IR),TNF-α, IL-1β and IL-6 at the end of the trial. Table S2. Blood glucose concentrations during the oral glucose tolerance tests (OGTTs) following the treatment for 6 weeks. Table S3. Blood glucose concentrations during the oral glucose tolerance tests (OGTTs) following the treatment for 8 weeks. Table S4. Blood glucose concentrations during the oral glucose tolerance tests (OGTTs) following the treatment for 10 weeks. Tables S5 and S6 TLR mRNA relative expression pattern of skeletal muscle. Table S7 and the original western blots Effect of WEM treatment on protein expression TLR1, TLR2, and the downstream transcription factors' expression. Table S8. The gene expression of IRS1 and InsR affected by WEM in adipose tissue. Table S9 and the original western blots Protein expression of IRS1 and InsR influenced by WEM in adipose tissue of diabetic mice. [file 12906_2019_2742_MOESM1_ESM.pdf]
